# Variability in the Precore and Core Promoter Regions of HBV Strains in Morocco: Characterization and Impact on Liver Disease Progression

**DOI:** 10.1371/journal.pone.0042891

**Published:** 2012-08-14

**Authors:** Bouchra Kitab, Abdellah Essaid El Feydi, Rajaa Afifi, Christian Trepo, Mustapha Benazzouz, Wafaa Essamri, Fabien Zoulim, Isabelle Chemin, Hanane Salih Alj, Sayeh Ezzikouri, Soumaya Benjelloun

**Affiliations:** 1 Laboratoire des Hépatites Virales, Institut Pasteur du Maroc, Casablanca, Morocco; 2 Service Médecine C, CHU Ibn Sina, Rabat, Morocco; 3 INSERM, Unité 1052, Lyon, France; 4 Laboratoire de Recherche sur les Lipoprotéines et l′Athérosclérose, Unité Associée au CNRST -URAC 34- Université Hassan II, Faculté des Sciences Ben M′sik, Casablanca, Morocco; St. Louis University, United States of America

## Abstract

**Background:**

Hepatitis B virus (HBV) is one of the most common human pathogens that cause aggressive hepatitis and advanced liver disease (AdLD), including liver cirrhosis and Hepatocellular Carcinoma. The persistence of active HBV replication and liver damage after the loss of hepatitis B e antigen (HBeAg) has been frequently associated with mutations in the pre-core (pre-C) and core promoter (CP) regions of HBV genome that abolish or reduce HBeAg expression. The purpose of this study was to assess the prevalence of pre-C and CP mutations and their impact on the subsequent course of liver disease in Morocco.

**Methods/Principal Findings:**

A cohort of 186 patients with HBeAg-negative chronic HBV infection was studied (81 inactive carriers, 69 with active chronic hepatitis, 36 with AdLD). Pre-C and CP mutations were analyzed by PCR-direct sequencing method. The pre-C stop codon G1896A mutation was the most frequent (83.9%) and was associated with a lower risk of AdLD development (OR, 0.4; 95% CI, 0.15–1.04; *p* = 0.04). HBV-DNA levels in patients with G1896A were not significantly different from the other patients carrying wild-type strains (*p* = 0.84). CP mutations C1653T, T1753V, A1762T/G1764A, and C1766T/T1768A were associated with higher HBV-DNA level and increased liver disease severity. Multiple logistic regression analysis showed that older age (≥40 years), male sex, high viral load (>4.3 log_10_ IU/mL) and CP mutations C1653T, T1753V, A1762T/G1764A, and C1766T/T1768A were independent risk factors for AdLD development. Combination of these mutations was significantly associated with AdLD (OR, 7.52; 95% CI, 4.8–8; *p*<0.0001).

**Conclusions:**

This study shows for the first time the association of HBV viral load and CP mutations with the severity of liver disease in Moroccan HBV chronic carriers. The examination of CP mutations alone or in combination could be helpful for prediction of the clinical outcome.

## Introduction

Hepatitis B virus (HBV) infection is a major public health problem, with approximately 360 million chronic carriers worldwide. These patients are at increased risk of developing advanced liver disease (AdLD), including liver cirrhosis (LC) and hepatocellular carcinoma (HCC), accounting for 0.5 to 1.2 million deaths annually [1], [2]. In Morocco, the prevalence of HBV infection is intermediate; ranging from 1.5% to 2.5% and the incidence of HCC was considered relatively rare, representing 6% of hepatic tumors [3]–[6]. 12.5% of HCC cases are attributable to HBV infection [7]. In addition, previous studies concerning HBV genotype distribution among Moroccan HBV carriers showed a predominance of genotype D, subgenotype D7 with limited circulation of genotype A, subgenotype A2 [8], [9].

In the natural history of chronic hepatitis B (CHB), hepatitis B e antigen (HBeAg) expression has been used clinically as an indicator of active HBV replication, and seroconversion from HBeAg to its antibody, anti-HBe, is usually characterized by a decrease in HBV replication, normalization of serum alanine aminotransferases (ALT) levels and remission of liver disease, which correspond to the so called “inactive HBV carrier state” [10]. Although the majority of patients remain for life time in an inactive carrier state, other HBeAg-negative subjects retain or redevelop active HBV replication associated with progressive liver damage [11]. This form of disease is referred as HBeAg-negative CHB (e-CHB) and is mostly associated with mutations in the pre-core (pre-C, nucleotides (nts) 1814–1900) and core promoter (CP, nts 1613–1849) regions of HBV genome [12]. The pre-C region contains the encapsidation signal, whose stability is important for the packaging of pregenomic messenger RNA (pg-mRNA) and for the priming of genomic replication [13]. In this region, HBeAg expression is abolished at the translational level by nonsense, frameshift, or initiation codon mutations. The most common mutation involves a G→A transition at position 1896 (G1896A), which creates a premature stop codon at codon 28, causing the termination of HBeAg translation without affecting the replicative ability of the virus [14]. The CP plays an essential role in HBV replication and morphogenesis directing the transcription of pg-mRNA and pre-C messenger RNA (pre-C mRNA). It is composed of two regions: the upper regulatory region (URR, nts 1613–1742) and the basal core promoter (BCP, nts 1742–1849) [15]. CP mutations down regulate HBeAg expression at the transcriptional level. The most frequent mutation is an A→T substitution at nt 1762 combined with G→A substitution at nt 1764 (A1762T/G1764A), which results in a decreased transcription of pre-C RNA by 50 to 70%, but enhanced viral replication *in vitro* [15]. Moreover, a relationship between pre-C/CP mutations and HBV genotypes has been reported. G1896A mutation was seen at a higher frequency in genotype D strains, while A1762T/G1764A double mutation was observed predominantly in genotype A strains [16].

The clinical significance of G1896A mutation is unclear, with reports of its association with severe forms of liver disease and others of its occurrence in a large number of patients who had inactive liver disease [17]–[20]. Moreover, other studies reported no association between G1896A and clinical outcome [21]–[23]. A1762T/G1764A double mutation has been frequently reported to be associated with the progression of liver disease and increased risk for HCC, but it has also been found in patients without AdLD [17], [21], [22], [24], [25]. Recently, additional mutations in the pre-C and CP regions, including G1899A, C1653T, T1753V, C1766T and T1768A have become increasingly recognized to be associated with severe clinical outcome and HCC development [21], [24], [26], [27].

HBeAg-negative chronic HBV infections are common in Morocco [5], [8]. However, up to now, no concrete data are available about the prevalence of mutations in the pre-C and CP regions and their relationship with the subsequent course of HBV-related liver disease. Our previous study focused only on pre-C G1896A and G1899A mutations by using selective oligonucleotide hybridization, and showed a predominance of these variants [28]. The present study was carried out to determine the prevalence of pre-C and CP mutations in a large cohort of HBeAg-negative patients and to evaluate their association with viral replication and severity of liver disease, based on direct-sequencing method.

## Materials and Methods

### Patients

The study included 186 treatment-naïve patients with HBeAg-negative chronic HBV infection, collected to the Medical Center of Biology at the Pasteur Institute of Morocco, Casablanca and admitted to Ibn Sina hospital, Rabat from November 2008 to July 2011. All patients were anti-HBe positive and negative for HCV, HDV and HIV antibodies.

These patients were further classified into three clinical groups: (1) Inactive HBV carriers (IC, *n* = 81) with persistently normal serum ALT level (≤40 IU/L) and low HBV-DNA level (<3.3 log_10_ IU/mL) during at least 6 months of follow up (2) Patients with active chronic hepatitis (ACH, *n* = 69) defined by persistent or intermittent elevation in ALT level (>40 IU/L) and HBV-DNA level (≥3.3 log_10_ IU/mL) for at least 6 months and histological signs of moderate/severe necroinflammation. (3) AdLD group (*n* = 36), including 22 patients with LC and 14 patients with HCC. LC was diagnosed by ultrasonography and clinical criteria indicating portal hypertension (ascites, esophageal varices, etc). The diagnosis of HCC was based on serum alpha-foetoprotein levels (AFP), imaging showing the characteristic features of HCC and/or when possible histological assessment of tissues samples. All patients gave informed consent for participation and the study was approved by Committee on Research Ethics of Institut Pasteur du Maroc. Collected serum samples were stored at −20°C until use.

### HBV-DNA quantification and Genotyping

Serum HBV-DNA was quantified by a fully automatic system (COBAS AmpliPrep/COBAS TaqMan, Roche Diagnostics) for HBV-DNA extraction and real-time PCR quantification. The quantification range of this assay was 1 to 9 log_10_ IU/mL. HBV genotypes and subgenotypes were determined by direct sequencing of HBV surface (S) gene and phylogenetic analysis, as described previously [8].

### HBV-DNA extraction, amplification and direct sequencing of Pre-C and CP regions

HBV-DNA was isolated from 140 µl serum by proteinase K digestion followed by extraction with phenol chloroform and then precipitation with ethanol. The obtained pellet was dissolved in 60 µl of 1X TE buffer (10 mmol/L Tris, 1 mmol/L EDTA).

The pre-C and CP regions were amplified by hemi-nested PCR using the primers 1609 (5′-CATGGAGACCACCGTGAAC-3′ [nts: 1609–1627], forward) and HBC4 (5′-CCCACCTTATGAGTCCAAGG-3′ [nts: 2476–2457]; reverse) in the first round and the primers 1609 and MD25A (5′CGAGAGTAACTCCACAGT-3′, [nts: 1953–1936]; reverse) in the second round as previously described [29], [30]. First-round PCR was performed with 5 µl of DNA extract in a 25 µl reaction mixture containing 1X Taq polymerase buffer, 1.5 mmol/L MgCl2, 200 µmol/L dNTPs, 25 pmol of each primers and 1U of Taq polymerase (Invitrogen, France). The PCR condition was: preheating at 95°C for 5 min, then 35 cycles of amplification (94°C for 45 s, 60°C for 1 min and 72°C for 1 min) and final extension at 72°C for 10 min. For the second-round PCR, 1.5 µl of the first-round product was reamplified using the same reaction mixture composition. The PCR condition was: preheating at 94°C for 2 min, then 35 cycles of amplification (94°C for 15 s, 58°C for 20 s and 72°C for 35 s) and final extension at 72°C for 4 min.

A PCR product of 345 base pairs (bp) was obtained and subsequently purified using the Exonuclease I/Shrimp Alkaline Phosphatase (GE Healthcare). The purified products were bidirectionally sequenced using the BigDye® Terminator Version 3.1 kit (Applied Biosystems, Foster City, CA, USA) and analyzed using an ABI PRISM 3130 automated sequencer (Applied Biosystems, Foster City, CA, USA).

### Pre-C and CP mutations analysis

We initially identified the consensus sequences of pre-C/CP regions for HBV genotype A from 49 reference sequences subgenotype A2 and for HBV genotype D from 162 genotype D reference sequences. The sequences were aligned using Clustal X v2.0 software and edited using BioEdit v7.0 program [31], [32]. A nucleotide with the highest frequency at each site in the regions pre-C/CP was defined as wild-type nucleotide. All reference sequences were isolated from asymptomatic carriers (blood donors or pregnant women) and their GenBank accession numbers were listed in Text S1. Nucleotide sequences generated in this work were analyzed and compared to consensus sequence of corresponding genotype in both directions using SeqScape®v2.5 software (Applied Biosystems). We analyzed BCP mutations in the functional region bound by nt 1742 and 1791 (implicated in the synthesis of pre-C RNA). URR and pre-C mutations were analyzed in the entire regions. The nucleotide mutation was defined by difference to the consensus sequence and dual signals (mixed type) were considered as a mutant type.

### Statistical analysis

All the statistical analyses were performed using Statistical Package for Social Sciences (SPSS) program (SPSS Inc., Chicago, IL, USA). One-way analysis of variance was conducted to compare mean quantitative values and the χ2 or Fischer's exact test for categorical variables. Independent predictors of AdLD were identified by multiple logistic regression analysis. All *p-*values were two sided and *p-*value<0.05 was considered as statistically significant.

## Results

### Characteristics of Patients

The baseline characteristics of patients according to clinical status are presented in Table 1. There were 122 (65.6%) men and 64 (34.4%) women, with a mean age of 41.3±12.2 years (range: 19–80 years). No statistical difference was observed for age and sex between IC and ACH groups (*p*>0.05). Compared to these two groups, the AdLD group had significantly higher proportion of patients who were men (*p*<0.0001) and older in age (*p* = 0.0001). HBV-DNA level was significantly lower in AdLD group than in ACH group (*p* = 0.029).

**Table 1:** Baseline characteristics of patients

Characteristics	Non-AdLD (*n* = 150)		AdLD (*n* = 36)
	IC (*n* = 81)	ACH (*n* = 69)	
**Sex ratio (Male/Female)** *	50/31 (1.6)	44/25 (1.7)	28/8 (3.5)
**Age (year, mean ± SD)** †	41.4±11.6	38.2±11.5	54.5±12
**ALT (IU/L, mean ± SD)** ‡	37.3±7.3	93.1±78.9	100.9±80.5
**HBV-DNA (log_10_ IU/mL)** §			
Median [min-max]	2.8 [1.5–3.4]	5.4 [3.7–9]	4.5 [3.9–6.5]
**HBV genotype, ** ***n*** ** (%)**			
Genotype A2¶	10 (12.3)	7 (10.1)	6 (16.7)
Genotype D^∥^	71 (87.6)	62 (89.8)	30 (83.3)
**HBV/D subgenotype, ** ***n*** ** (%)**			
D1**	14 (17.3)	16 (23.2)	11 (30.5)
D2	1 (1.2)	0 (0)	0 (0)
D4	0 (0)	0 (0)	1 (2.8)
D7 ††	56 (69.1)	45 (65.2)	15 (41.7)
ND	0 (0)	1 (1.4)	3 (8.3)

Abbreviations: IC = inactive HBsAg carriers; ACH = active chronic hepatitis; AdLD = Advanced liver disease; ALT = alanine aminotransferase; IU = international units; ND = not determined.

* AdLD *vs* non-AdLD, *p* = 0.0001

† AdLD *vs* non-AdLD, *p*<0.0001

‡ IC *vs* ACH, *p*<0.001; IC *vs* AdLD, *p*<0.0001; ACH *vs* AdLD, *p* = 0.735

§ IC *vs* ACH, *p*<0.0001; IC *vs* AdLD, *p*<0.0001; ACH *vs* AdLD, *p* = 0.029

¶ IC *vs* ACH, *p* = 0.798; IC *vs* AdLD, *p* = 0.566; ACH *vs* AdLD, *p* = 0.361

∥ IC *vs* ACH, *p* = 0.798; IC *vs* AdLD, *p* = 0.361; ACH *vs* AdLD, *p* = 0.361

** IC *vs* ACH, *p* = 0.416; IC *vs* AdLD, *p* = 0.142; ACH *vs* AdLD, *p* = 0.483

†† Non-AdLD *vs* AdLD, *p* = 0.007

The distribution of HBV genotypes was genotype D in 163 (87.6%) patients and genotype A in 23 (12.4%) patients. All genotype A strains belonged to subgenotype A2. Among genotype D strains, subgenotype D7 was the most frequent (116/163, 71.2%), followed by subgenotypes D1 (41/163, 25.1%), D2 (1/163, 0.6%) and D4 (1/163, 0.6%). Four HBV/D strains could not be assigned to any described subgenotype. There was no significant difference in the distribution of genotypes A2 and D between the three clinical groups (*p*>0.05). The prevalence of subgenotype D1 increased with disease progression, although the difference did not reach statistical significance between the different clinical groups (OR = 2.11; 95% CI [4.8–8]; *p* = 0.183). HBV subgenotype D7 was significantly more predominant in non-AdLD group than in AdLD group (OR = 0.347; 95% CI [0.154–0.770]; *p* = 0.007) (Table 1).

### Prevalence of Pre-C and CP mutations

The analysis of 186 HBV-DNA sequences bearing the pre-C, URR and BCP regions was shown in Table 2. At least one mutation was found in 176 (94.6%), 106 (57%), 151 (81.2%) patients in pre-C, URR and BCP regions, respectively.

**Table 2:** Prevalence of mutations in the Pre-C and CP regions among different clinical groups

Mutation frequency	Non-AdLD (*n* = 150)		AdLD (*n* = 36)	Overall (*n* = 186)
	IC (*n* = 81)	ACH (*n* = 69)		
**Pre-C mutations, ** ***n*** ** (%)**				
TIM	6 (7.4)	1 (1.4)	0 (0)	7 (3.8)
G1896A*	70 (86.4)	60 (86.9)	26 (72.2)	156 (83.9)
G1899A†	37 (45.7)	38 (55.1)	23 (63.9)	98 (52.7)
OM	14 (17.3)	8 (11.6)	8 (22.2)	30 (16.1)
All mutated sequences	77 (95.1)	65 (94.2)	34 (94.4)	176 (94.6)
**URR mutations, ** ***n*** ** (%)**				
T1636G	7 (8.6)	8 (11.6)	3 (8.3)	18 (9.7)
C1637A	12 (14.8)	6 (8.7)	3 (8.3)	21 (11.3)
C1653T‡	3 (3.7)	8 (11.6)	15 (41.7)	26 (14)
OM	18 (22.2)	20 (29)	10 (27.7)	48 (25.8)
All mutated sequences	40 (49.4)	44 (63.7)	22 (61.1)	106 (57)
**BCP mutations, ** ***n*** ** (%)**				
T1753V§	9 (11.1)	24 (34.7)	20 (55.5)	53 (28.5)
A1757G	18 (22.2)	15 (21.7)	7 (19.4)	40 (21.5)
A1762T/G1764A¶	9 (11.1)	23 (33.3)	23 (63.9)	55 (29.5)
G1764T/C1766G	17 (21)	13 (18.8)	4 (11.1)	34 (18.3)
C1766T/T1768A^∥^	0 (0)	11 (15.9)	13 (36.1)	24 (13)
OM	19 (23.4)	16 (23.2)	6 (16.7)	41 (22)
All mutated sequences	52 (64.2)	66 (95.6)	33 (91.6)	151 (81.2)

Abbreviations: IC = inactive HBsAg carriers; ACH = active chronic hepatitis; AdLD = advanced liver disease; OM = others mutations; TIM = translation initiation mutation (nts 1814–1819).

* IC *vs* ACH, *p* = 0.189; AdLD *vs* non-AdLD, *p* = 0.04

† IC *vs* ACH, *p* = 0.251; IC *vs* AdLD, *p* = 0.03; ACH *vs* AdLD, *p* = 0.114

‡ IC *vs* ACH, *p* = 0.251; IC *vs* AdLD, *p*<0.0001; ACH *vs* AdLD, *p* = 0.0005

§ IC *vs* ACH, *p* = 0.001; IC *vs* AdLD, *p*<0.0001; ACH *vs* AdLD, *p* = 0.02

¶ IC *vs* ACH, *p* = 0.0003; IC *vs* AdLD, *p*<0.0001; ACH *vs* AdLD, *p* = 0.003

∥ ACH *vs* AdLD, *p* = 0.014

In the pre-C region, G1896A mutation occurred the most frequently (83.9%). G1899A mutation was also frequent (52.7%) and was mostly accompanied by G1896A in 87 patients (46.8%). Mutations affecting the initiation of translation of pre-C gene (TIM) were found in 7 (3.8%) patients, including A1814C (three ICs), T1815C (one IC), T1815A (one patient with ACH) and C1817T (two ICs). No patients with TIM had concomitant G1896A mutation.

In the CP region, eleven frequent mutations were found: C1653T was the most common in the URR (14%) followed by C1637A (11.3%) and T1636G (9.7%). In the BCP, G1764A was the most frequent (72/186, 38.7%), followed by A1762T (59/186, 31.7%), T1753V (28.5%), A1757G (21.5%), C1766G (48/186, 26%), C1766T (41/186, 22%), G1764T (35/186, 18.8%) and T1768A (29/186, 15.6%). G1764A and A1762T were frequently found together in 55 patients (29.5%). Likewise, T1768A was frequently present in association with C1766T in 24 (12.4%) patients. Thus, G1764A/A1762T and C1766T/T1768A were considered as mutational patterns.

### Prevalence of Pre-C and CP mutations according to HBV genotypes

**Figure 1:**
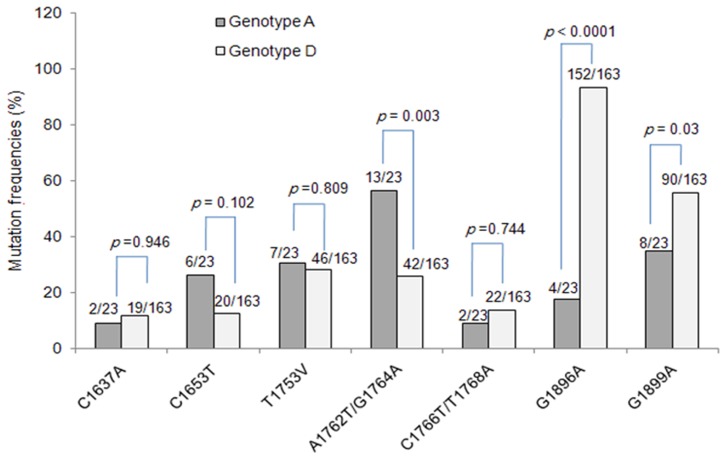
Prevalence of Pre-C and CP mutations according to HBV genotypes A and D.

As shown in Figure 1, G1896A and G1899A were more common in genotype D than in genotype A strains (*p*<0.001, *p* = 0.03, respectively) while A1762T/G1764A was more frequent in genotype A than in genotype D strains (*p* = 0.003). All nucleotides sequences corresponding to genotypes D had a thymidine “T” at nt 1858, whereas all genotype A sequences had a cytosine “C” at nt 1858, except four cases harboring G1896A, in which concomitant C1858T mutation occurred. Moreover, no significant difference was observed between genotype A and D regarding the prevalence of mutations C1637A (*p* = 0.946), C1653T (*p* = 0.102), T1753V (*p* = 0.809), and C1766T/T1768A (*p* = 0.744). The mutations T1636G, A1757G and G1764T/C1766G were exclusively found in genotype D strains. The TIMs were found only in genotype A strains.

### Prevalence of Pre-C and CP mutations according to clinical status

The prevalence of pre-C and CP mutations in relation to the clinical status of patients was shown in Table 2. G1896A mutation occurred significantly more frequently in non-AdLD group than in AdLD group (*p* = 0.04). The prevalence of G1899A mutation was significantly higher in AdLD group than in ICs (*p* = 0.03).

With regard to the CP mutations, six were associated with the clinical status of patients: C1653T, G1764A, A1762T, T1753V, C1766T and T1768A. The prevalence of C1653T was significantly higher in AdLD group than in IC (*p*<0.0001) and ACH group (*p* = 0.0005). The prevalence of T1753V and A1762T/G1764A increased significantly with disease progression (IC *vs.* ACH, *p* = 0.001 and ACH *vs.* AdLD, *p* = 0.02; IC *vs.* ACH, *p* = 0.0003 and ACH *vs.* AdLD, *p* = 0.003, respectively). C1766T/T1768A was found only among ACH and AdLD groups, with a significantly higher prevalence in AdLD group (*p* = 0.014).

### Pre-C and CP mutations in relation to clinical features of patients

The significance of pre-C and CP mutations in relation to the clinical features of patients was illustrated in Table 3. There were no significant differences between patients with and without G1896A regarding mean age (*p* = 0.56) and HBV-DNA level (*p* = 0.84), but compared to patients infected with the wild-type virus, patients carrying G1896A mutation had a lower ALT level although the difference did not reach statistical significance (*p* = 0.06). In addition, patients harboring strains with G1899A, C1653T, T1753V, A1762T/G1764A and C1766T/T1768A mutations were significantly older than those without these mutations (*p*≤0.01). Both elevated ALT and high HBV-DNA levels were significantly associated with the presence of T1753V, A1762T/G1764 and C1766T/T1768A mutations (*p*<0.001).

**Table 3:** Comparison of clinical features of patients according to Pre-C and CP Mutations

Mutation	Age (year)(mean ± SD)	ALT (IU/L)(mean ± SD)	HBV-DNA(Log_10_ IU/mL)*
**G1896A**			
Presence (*n* = 156)	42.3±13.1	55.5±47	3.8 [1.5–7]
Absence (*n* = 30)	43.8±12	73.2±46.4	3.9 [2.8–9]
*p*-value	0.56	0.06	0.84
**G1899A**			
Presence (*n* = 98)	45.2±12.6	70.4±71.2	4.3 [2.6–9]
Absence (*n* = 88)	39.6±12.5	49.1±28.3	3.7 [1.65–7.4]
*p*-value	0.004	0.07	0.23
**C1653T**			
Presence (*n* = 26)	48.5±14.5	62±39.7	3.9 [2.8–9]
Absence (*n* = 160)	41.6±12.4	58.6±57.4	3.8 [1.5–7.8]
*p*-value	0.017	0.752	0.823
**T1753V**			
Presence (*n* = 53)	46.8±10.9	94.1±95.5	5.2 [2.4–9]
Absence (*n* = 133)	40±11	49.2±31.2	3.7 [1.5–7.4]
*p*-value	0.001	0.0005	0.006
**A1762T/G1764A**			
Presence (*n* = 55)	45.3±13.9	92.8±90.3	5.3 [2.6–9]
Absence (*n* = 131)	39±12.4	47.1±31	3.9 [1.5–6.7]
*p*-value	0.002	0.0001	0.002
**C1766T/T1768A**			
Presence (*n* = 24)	48.2±15	83.2±46.4	5.6 [3.7–9]
Absence (*n* = 162)	41±11.8	55.5±47.1	3.7 [1.5–7]
*p*-value	0.018	0.001	<0.0001

* median range [min-max].

**Figure 2:**
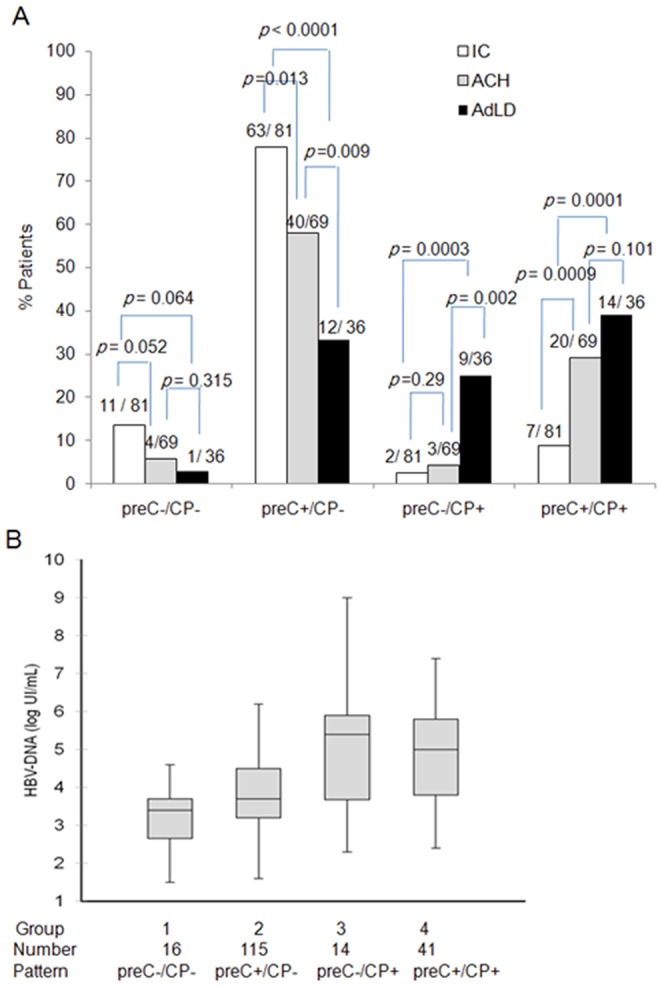
Association of the PreC/CP mutational patterns with clinical status (A) and HBV-DNA level (B). Four patterns (preC−/CP−, preC+/CP−, preC−/CP+, and preC+/CP+) were defined based on the presence (+) or absence (−) of pre-C mutation G1896A and CP A1762T/G1764A double mutation. For HBV-DNA levels, the data are presented as box plots, illustrated as the median (horizontal line) and the range from the 25th to the 75th percentiles. Group 1 *vs* 2, *p* = 0.09, Group 3 *vs* 1, *p* = 0.009; Group 3 *vs* 2, *p* = 0.02; Group 3 *vs* 4, *p* = 0.627; Group 4 *vs* 2, *p* = 0.02.

Further combined analysis of common pre-C (G1896A) and CP (A1762T/G1764A) mutations was performed in all patients. Accordingly, four patterns were identified: with neither mutation (preC−/CP− [n = 16, 8.6%]), with pre-C mutation only (preC+/CP− [n = 115, 61.8%]), with CP mutation only (preC−/CP+ [n = 14, 7.5%]), and with both mutations (preC+/CP+ [n = 41, 22%]). There were significantly more preC−/CP+ viruses in AdLD group than in IC (*p* = 0.0003) and ACH group (*p* = 0.002). Also, preC+/CP+ viruses were frequently found in ACH and AdLD groups than in IC group (*p* = 0.0009, *p* = 0.0001, respectively). In contrast, preC+/CP− viruses were frequently detected in IC group compared with ACH (*p* = 0.013) and AdLD groups (*p*<0.0001) (Figure 2A). In addition, HBV-DNA levels were significantly higher in patients infected with preC−/CP+ or preC+/CP+ viruses compared to those with preC+/CP− and preC−/CP− viruses (Figure 2B).

### Multivariate analysis of factors associated with AdLD development

To determine the independent predictive factor for the development of AdLD, multiple logistic regression analysis was performed. It was found that older age (≥40 years), male sex, high serum HBV-DNA level (>4.3 log_10_ IU/mL) and the presence of mutations C1653T, T1753V, A1762T/G1764A and C1766T/T1768A were independent risk factors predictor of AdLD development, whereas G1896A was associated with a decreased risk of AdLD development (Table 4).

**Table 4:** Multiple logistic regression analysis for factors independently associated with AdLD development

	*n* = 186	
Factor	OR (95% CI)	*p*-value
**Age**		
<40 years	1	
≥40 Years	14.20 (3.10–89.28)	<0.001
**Sex**		
Female	1	
Male	5.87 (2.35–15.13)	<0.001
**HBV-DNA (log_10_ IU/mL)**		
>3.3	1	
3.3–4.3	2.46 (0.75–8.04)	0.09
>4.3	3.08 (1.03–9.49)	0.029
**HBV genotype**		
D	1	
A	1.56 (0.50–4.67)	0.401
**G1896A**		
Presence	1	
Absence	0.40 (0.15–1.04)	0.04
**G1899A**		
Absence	1	
Presence	1.77 (0.79–4.01)	0.142
**C1653T**		
Absence	1	
Presence	9.02 (3.35–23.64)	<0.0001
**T1753V**		
Absence	1	
Presence	4.26 (1.87–9.79)	0.0001
**A1762T/G1764A**		
Absence	1	
Presence	5.57 (2.41–13.01)	<0.0001
**C1766T/T1768A**		
Absence	1	
Presence	7.14 (2.61–19.74)	<0.0001

### Multiple mutations in the CP and increased risk for AdLD

**Figure 3:**
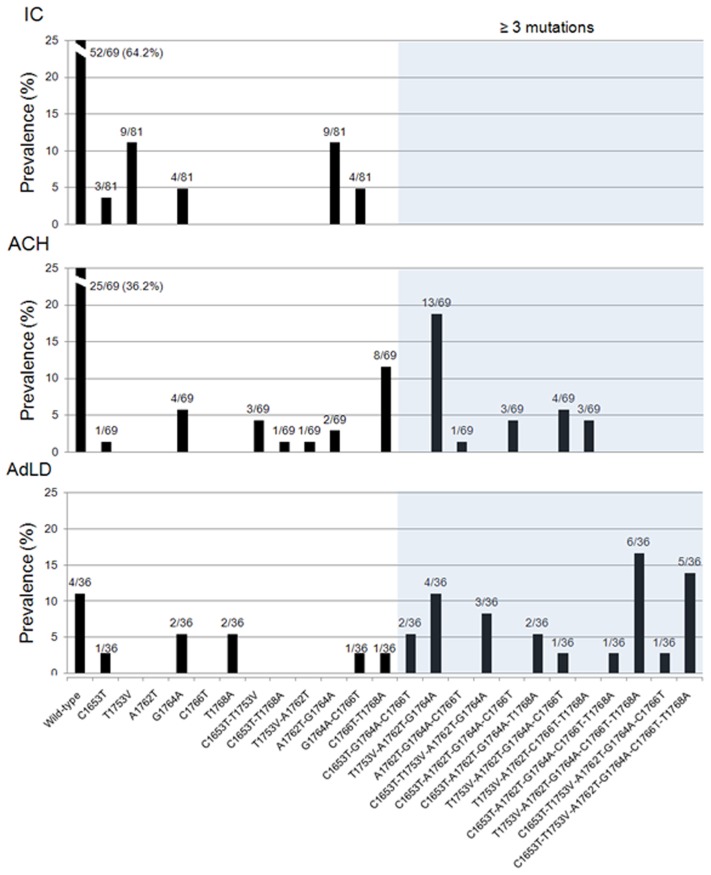
Frequency of multiple mutations in the CP region among patients at different stages of liver disease. Wild-type represents strains without mutations in indicated sites.

It has been previously reported that combined mutations in the CP may lead to more severe liver disease [26], [27]. Therefore, we investigated the frequency of the combination of the six CP mutations independently associated with AdLD (C1653T, T1753V, A1762T, G1764A, C1766T, and T1768A) in the 3 clinical groups (Figure 3). A combination of three or more mutations was found in 24 (34.8%) and 25 (69.4%) patients with ACH and AdLD, respectively, whereas none of IC had such mutational patterns (IC *vs.* ACH, *p*<0.0001; ACH *vs.* AdLD, *p* = 0.001). Triple mutations were found in 14 ACH and 6 AdLD patients, with T1753V/A1762T/G1764A, the most frequent. Quadruple mutations were observed in 10 patients with ACH, and 6 patients with AdLD, whereas quintuple and sextuple mutations were observed exclusively among 13 patients with AdLD. Using multivariate analysis, carriage of more than four mutations was significantly associated with AdLD (OR, 7.52; 95% CI, 4.8–8; *p*<0.0001).

## Discussion

To our knowledge, this is the first study that assessed the prevalence of mutations in the pre-C and CP regions of HBV genome and to evaluate their relationship with clinical manifestations among Moroccan HBeAg-negative patients. The majority of patients were infected with HBV genotype D (87.6%), subgenotype D7 (71.2%), and only 12.4% had genotype A/subgenotype A2. These results confirmed previous genotyping efforts in Morocco [8], [9]. HBV genotype A2, prevalent in Europe, was probably introduced in Morocco through population movements from Europe to our country. This genotype has been reported to be associated with higher rate of the transition into inactive carrier stage after HBeAg seroconversion compared to genotype D [33]. In the present study, no difference was seen in the distribution of genotypes A2 and D between patients with inactive and severe liver disease, which is consistent with previous findings [34]. Moreover, a significant higher frequency of subgenotype D7 was found among patients without AdLD compared to those with AdLD, suggesting an association of this subgenotype with more benign course of liver disease.

The analysis of nucleotide sequences of the pre-C and CP regions showed that the pre-C stop codon G1896A mutation was the major cause of HBeAg negativity, detected in 83.9% of patients. This prevalence was similar to those found among HBeAg-negative patients in our earlier study (86.6%) [28], but lower than those reported in another Moroccan study, in which the HBeAg status of patients has not been mentioned (61.4%) [9]. Moreover, comparable frequencies of G1896A have been reported among HBeAg-negative carriers in Turkey (85%) and Algeria (80%) [23], [35]. The CP A1762T/G1764A double mutation that diminishes HBeAg production was found in 29.5% of patients. This prevalence was relatively similar to those reported in other Moroccan study (22.6%) [9], and lower than those reported in Algeria (60%) and Tunisia (53.6%) [35], [36]. The reason that may account for the higher prevalence of A1762T/G1764A double mutation in these countries compared to Morocco is unclear. We suggest that the host factors may influence the occurrence of this mutation, as has been reported previously [37]. Interestingly, it has been reported that the mechanism that prevent HBeAg expression was different between genotypes A2 and D, explaining the fact that patients infected with genotype A2 were frequently HBeAg positive and showed later HBeAg clearance compared to those infected with genotype D [38]. In the present study, 93.2% of genotype D strains had pre-C stop codon G1896A mutation, whereas only four genotype A2 strains had this mutation. This result was consistent with previous data showing that G1896A mutation was the strongest factor of HBeAg clearance in HBV/D-infected individuals [38], [39]. The basis of the relation between G1896A mutation and HBV genotypes is due to the base pairing of the stem loop structure of the encapsidation signal (ε), essential for viral replication. The nt 1896 is located opposite to nt 1858 in the stem loop, therefore, G1896A is restricted to genotypes that have T at nt 1858 (non-A genotypes), since its stabilizes the encapsidation signal, and occurred rarely in genotypes that have C at nt 1858 (genotype A), which is line with our findings [16]. In contrast to genotype D strains, a higher frequency of the CP A1762T/G1764A double mutation was detected in genotype A2 strains. This finding was supported by the results of a previous study suggesting that the double mutation A1762T/G1764A would be associated with the loss of HBeAg in HBV/A2 carriers [38].

The clinic-pathologic importance of the common G1896A and A1762T/G1764A mutations in the course of HBV infection has been extensively studied. In the present work, multivariate analysis showed that G1896A mutation was associated to a lower risk of AdLD compared to the wild-type virus. This result seems to be in line with two recent studies reporting a negative association between G1896A mutation and HCC [40], [41]. Similarly, another study reported that hepatic inflammation occurred more frequently in patients with the wild type than those with G1896A mutation [42]. No significant difference was found in the HBV-DNA level between our patients with and without G1896A, suggesting that G1896A had no effect on HBV replication and supporting previous *in vivo* and *in vitro* studies [37], [43], [44], but contradicted others reporting that G1896A mutation may enhance HBV replication by increasing the encapsidation signal stability [18]. All these results led us to speculate that persistent HBV replication after HBeAg seroconversion occurs independently of G1896A, with no pathogenic role of this mutation in the subsequent course of liver disease. In contrast, we found that the prevalence of A1762T/G1764A double mutation increased significantly with disease progression from IC to AdLD state, implicating this mutation in disease severity. Using multivariate analysis, we found that A1762T/G1764A mutation was an independent risk factor for AdLD development, which is agreement with several studies [17], [21], [22], [24]. The mechanism by which A1762T/G1764A increased HBV related pathogenesis is not fully understood. Functional studies demonstrated that A1762T/G1764A was associated with the upregulation of viral replication by creating a hepatocyte nuclear factor-1 (HNF-1) binding site or by reducing pre-C expression and concomitantly increasing pg-mRNA level, which result in increased HBV virulence [15]. In agreement with these data, we found that patients carrying A1762T/G1764A double mutation have significantly higher HBV-DNA levels compared to those with wild-type virus. In addition, a recent *in vitro* study revealed that A1762T/G1764A double mutation was associated with more cytoplasmic localization of intracellular hepatitis B core antigen (HBcAg) than wild-type strain [45]. Because cytoplasmic localization of HBcAg is closely related to active necroinflammation of hepatocytes [46], these data may partly explain the association between the emergence of A1762T/G1764A double mutation and liver damage. Furthermore, the CP overlaps with the coding sequence of HBx protein, which functions as a transcriptional *trans*-activator of viral genes and cellular genes encoding proteins regulating cell growth, DNA repair and apoptosis [47]. Therefore, mutations in the CP may result in amino acid (aa) changes in the HBx protein. The A1762T/G1764A double mutation changes two codons in HBx protein (K130M and V131I). In addition, A1762T mutation introduced a translation initiation site ATG at the C-terminal region of HBx protein [48]. Functional studies revealed that HBx mutations, especially at the C-terminal region lead to different regulatory activities in cell proliferation and transformation contributing to carcinogenesis [27], [48]. The role of the changes induced by A1762T/G1764A mutation on HBx protein is another important issue to consider in future studies.

The co-existence of A1762T/G1764A and G1896A mutations was observed in 22% of our patients. Previously, it has been suggested that the impact of A1762T/G1764A on viral replication may be modulated by G1896A [49]. In variance with this suggestion, we observed that the presence of A1762T/G1764A occurred more frequently in ACH and AdLD cases and was associated with higher HBV-DNA levels irrespective of pre-C 1896 status. Interestingly, we found that HBV sequences with G1896A alone were significantly more prevalent among IC than patients with ACH and AdLD. Our result was supported by the findings of previous studies reporting a significant link between G1896A mutation and inactive HBV carrier state [19], [20]. As it has been previously reported that A1762T/G1764A appears earlier than G1896A during the course of HBV infection [50], [51], we suggest that the carriage of G1896A mutation in the absence of A1762T/G1764A double mutation, is beneficial for maintaining an inactive disease status. We can also explained the ACH and AdLD cases found in G1896A-associated disease by the fact that G1896A has been acquired after the liver damage established by A1762T/G1764A. Further longitudinal studies are needed to clarify this observation.

Besides A1762T/G1764A double mutation, other mutations in the CP, including T1753V, C1653T and C1766T/T1768A, appear to be implicated in disease severity in Moroccan HBV carriers as reflected by their higher frequencies among patients with AdLD compared to IC and ACH patients. Multivariate analysis indicated that these mutations were independent risk factors for AdLD development, which is compatible with previous findings [21], [26], [27], [41]. The biological mechanisms that explain the association of C1653T, T1753V, C1766T and T1768A mutations with the pathogenesis of HBV infection are less well defined. C1653T mutation changes the box-alpha binding site for C/EBP and related factors into a perfect palindromic sequence, which could enhance the activity of the core promoter and HBV replication. In addition, this mutation changes the aa 94 (H94Y) in the HBx protein, which affect the transactivation effects of this protein [15]. In the present work, no difference was observed in the viral load between patients with and without C1653T, in agreement with some *in vitro* studies [15]. Thus, the relationship between this mutation and severity of liver disease is not mediated by an increase in HBV replication but probably to direct pathogenic or oncogenic effects of this mutation. The T1753V mutation had earlier been associated with HCC development among Mongolian patients with genotype D [39]. Regarding the biological aspects, it was suggested that T1753V lead to the enhancement of transactivation and antiproliferation activity of HBx protein by altering HBx aa 127 (I127T/N/S) and enhancement of virus replication leading to persistent infection and higher frequency of HBV-DNA integration events into the human genome [52]. For the double mutation C1766T/T1768A, it has been described as an independent factor predictive of cirrhosis in HBeAg-negative patients and associated with a higher replication phenotype by increasing the encapsidation of pg-mRNA [27], [41], [53]. Our current results support these findings and suggest that C1766T/T1768A associated with high HBV-DNA levels may induce severe liver injury and increase the risk of AdLD. Moreover, T1768A result in aa changes at HBx codon 132 (F132Y), that have been showed to play a synergetic role with K130M and V131I introduced by A1762T/G1764A for leading to carcinogenesis [27].

An interesting finding in this study is that the development of progressive liver disease was significantly associated with increased number of mutations in the CP. Of the six mutations pathologically implicated, C1653T, T1753V, A1762T, G1764A, C1766T and T1768A, a combination of three or more mutations was more likely associated with ACH and AdLD stages. Also, the presence of quintuple or sextuple mutations in the CP was especially observed among patients with AdLD. This frequent occurrence of CP multiple mutations in AdLD patients may in part be explained by the older age of these patients associated with longer duration of HBV infection, and therefore, increased risk for accumulation of mutations. Interestingly, it has been reported that the CP mutations accumulate in a certain order: A1762T/G1764A emerge first, being detectable approximately 10 years before the diagnosis of HCC followed by C1653T and finally, T1753V, C1766T or T1768A mutations [21], [26]. Therefore, A1762T/G1764A may serve as a useful biomarker for progressive liver damage. Another important observation is that in ICs harboring C1653T, T1753V and A1762T/G1764A, these mutations were not found in combination. Also C1766T and T1768A were mostly found alone among ACH patients and mostly accompanied by C1653T, T1753V and A1762T/G1764A in AdLD patients. On the basis of these data, we speculate that these combined mutations had an additive role in HBV pathogenesis and development of AdLD. *In vitro* studies have demonstrated a gradual enhancement of HBV replication when mutations at positions 1753, 1766 and 1768 are added to A1762T/G1764A double mutation [43], [50]. However, because of the very low number of cases with A1762T/G1764A alone, it could be difficult to establish the effect of combined CP mutations on HBV replication.

In conclusion, our results showed that the pre-C G1896A mutation is the major cause of HBeAg negativity in Moroccan chronic HBV carriers, with probably a beneficial role in the course of liver disease. The persistence of HBV replication and the severity of liver damage are attributed to A1762T/G1764A double mutation and associated mutations C1653T, T1753V and C1766T/T1768A in the CP. The A1762T/G1764A double mutation might be used as a biomarker for the prediction of future disease activity and might be helpful in the identification of patients that should be regularly followed for HBV-DNA level and criteria of AdLD or require therapeutic intervention.

## Supporting Information

Text S1 Genotypes/Subgenotypes and GenBank accession numbers for the HBV reference sequences used in this study.
(DOC)
