# Supplementary material for: Variability in the Precore and Core Promoter Regions of HBV Strains in Morocco: Characterization and Impact on Liver Disease Progression
Source: PLoS One. 2012 Aug 14;7(8):e42891. doi: 10.1371/journal.pone.0042891 (PMC3419231; doi:10.1371/journal.pone.0042891)
Supplement: Text S1 — Genotypes/Subgenotypes and GenBank accession numbers for the HBV reference sequences used in this study. (DOC) [file pone.0042891.s001.doc]

**Genotype D (n=162 sequences):**

Subgenotype D1 (n=62): AB104709, AB104712, FJ904399, FJ904412, FJ904420, FJ904443, AB090269, GU456684, GU456683, GU456682, GU456681, GU456680, GU456679, GU456678, GU456677, GU456676, GU456675, GU456674, GU456673, GU456672, GU456671,GU456670, GU456669, GU456668, GU456667, GU456666, GU456665, GU456664, GU456663, GU456662, GU456660, GU456661, GU456659, GU456658, GU456657, GU456656, GU456655, GU456654, JF754635, FJ904446, FJ904445, FJ904432, FJ904431, FJ904429, FJ904427, FJ904426, FJ904424, FJ904421, FJ904418, FJ904415, FJ904402, AB222710, AB222711, AB222712, AB222713, AF280817, AY721606, AY721607, AY721608, AY721609, AY721610, AY721611

Subgenotype D2 (n=35) : AB078033, AB090270, AB210820, AB109475, AB109476, AB109477, GU456635, JF754621, JF754597, GQ924652, GQ477457, GQ477456, GQ477455, GQ477454, GQ477453,GQ477452, EU594403, EU594402, EU594400, EU594410, EU594409, EU594416, EU594415, EU594428, EU594399, EU594432, EU594431, EU594408, EU594407, EU594430, EU594425, EU594423, EU594422, EU594421, EU594405

Subgenotype D3 (n=10) : AY233291, AY233293, AY233294, AY233295, AY233296, JF754625, EU594382, EU594436, EU594434, DQ111987, DQ315776

Subgenotype D4 (n=3): AB033559, AB048702, AB048703

Subgenotype D5 (n=15): AB033558, GQ205389, GQ205388, GQ205387, GQ205386, GQ205385, GQ205384, GQ205383, GQ205382, GQ205381, GQ205380, GQ205379, GQ205378, GQ205377, DQ315779, DQ315780

Subgenotype D6 (n=3): AB493846, AB493845, AB493848

Subgenotype D7 (n=32): FJ904400, FJ904401, FJ904403, FJ904404, FJ904405, FJ904407, FJ904408, FJ904409, FJ904410, FJ904413, FJ904414, FJ904416, FJ904417, FJ904419, FJ904425, FJ904428, FJ904430, FJ904433, FJ904435, FJ904436, FJ904437, FJ904438, FJ904439, FJ904440, FJ904441, FJ904442, FJ904444, FJ904447, FJ904394, FJ904395, FJ904397, FJ904398

**Genotype A2 (n=49 sequences):**

FJ904434, GQ477504, GQ477503, GQ477502, GQ477501, GQ477500, GQ477499, GQ477498, GQ477497, GQ477496, GQ477495, GQ477494, GQ477493, GQ477492, GQ477491, GQ477490, GQ477489, GQ477488, GQ477487, GQ477486, GQ477485, GQ477484, GQ477483, GQ477482, GQ477481, GQ477480, GQ477479, GQ477478, GQ477477, GQ477476, GQ477475, GQ477474, GQ477473, GQ477472, GQ477471, GQ477470, GQ477469, GQ477468, GQ477467, GQ477466, GQ477465, GQ477464, GQ477463, GQ477462, GQ477461, GQ477460, AY233280, AY233286, FJ904411, X02763
